# Mental health status and associated contributing factors among the Hakka elderly in Fujian, China

**DOI:** 10.3389/fpubh.2022.928880

**Published:** 2022-07-22

**Authors:** Xiaojun Liu, Fengyu Liu, Wenqian Ruan, Yating Chen, Shuming Qu, Wenxin Wang

**Affiliations:** ^1^Department of Health Management, School of Public Health, Fujian Medical University, Fuzhou, China; ^2^Department of Public Administration, Law School/Institute of Local Government Development, Shantou University, Shantou, China

**Keywords:** the Hakka elderly, mental health, socio-demographic determinants, Symptom Checklist-90-R, cross-sectional survey, China

## Abstract

**Purpose:**

Little is known about the mental health of the Hakka elderly. This study explores the status of, and factors associated with mental health among Hakka elderly populations from Fujian, China.

**Methods:**

This is a cross-sectional, community-based survey study containing a total of 1,262 valid samples. The Chinese version Symptom Checklist-90-R (SCL-90-R) was used to assess the mental health status of the Hakka elderly. We used *t*-tests to compare the differences for 10 dimensions of SCL-90-R scores between the Chinese national norm and the Hakka elderly. Univariate and multivariate analysis were performed by using linear regression analysis to identify the main socio-demographic factors that were most predictive of the total score of SCL-90-R in the Hakka elderly.

**Results:**

The scores of somatization (1.78 ± 0.55 vs. 1.40 ± 0.46, *P* < 0.001) and phobic anxiety (1.21 ± 0.36 vs. 1.17 ± 0.31, *P* < 0.001) for the Hakka elderly in Fujian appeared to be significantly higher than the Chinese norm. The higher total scores of SCL-90-R were found among females (β = 0.030, *P* = 0.044), widowed persons (β = 0.053, *P* = 0.021), those with parent(s) alive (β = 0.047, *P* = 0.019), and those with poorer self-rated health status (β = 0.110, *P* < 0.001). The lower total scores of SCL-90-R were found among those who were currently living in town, those with lower education level, those with higher average annual household incomes, and those who were living with spouse or children.

**Conclusion:**

The worse mental health conditions of the Hakka elderly in somatization and phobic anxiety were detected. The overall mental health status was shown to be worse among females, widowed persons, those who were living in village, those with lower education, and those with father or/and mother alive.

## Introduction

So far, many studies have been focused on the physical health of the elderly (1–3). With the increase of age, the deterioration of physical function will lead to multiple diseases and physical problems, such as non-communicable diseases (4). To some extent, with the development of modern medical technology, these diseases have been well-controlled. However, health is not only the absence of physical defects and diseases, but also a state of complete psychological and social adaptation. Compared with physical health, mental health is relatively easier to be neglected (5, 6). In China, the prevalence of mental illness like anxiety, depression appears to be increasing (6, 7). There is an urgent need for more attention from all walks of life. In addition, self-stigma and feelings of shame, deficiency of mental health care, imperfection of legal aid has caused obstructions of mental health treatment and management at present in China (8).

According to World Health Organization (WHO), over 20% of adults aged 60 and over suffer from a mental or neurological disorder (9). In China, the elderly with psychological disorders account for 27.8% of total population, and about one-third of the elderly suffer from depression in 2015 (6, 10, 11). In recent years, the mental health issues of the elderly are more serious due to the following reasons: first, with the rapid urbanization and “one child policy” o a large number of the elderly migrate to cities to take care of the younger generation (such as the grandchildren), posing a threat to the mental health of the elderly due to the poor social adaptability (6, 7, 11). While these who do not migrate to cities with their children are left behind, leaving more and more “empty nesters” e who are easier to suffer from mental illness, like loneliness (12). In addition, the proportion of elderly people having one or more chronic diseases is on the rise to 75% (13), and many physical chronic diseases could lead to mental illness (14, 15). Previous studies have shown that Chinese are more likely to avoid or delay seeking help for mental illness for some cultural reasons (16, 17). Therefore, more attention should be paid in mental health issues among elderly residents.

At present, there have been many reports about the mental problems targeting different groups of elderly. Trinh et al. reviewed mental health issues in racial and ethnic minority elderly (18), and concluded that racial and ethnic minority elders are preferentially vulnerable to mental health problems and often experience a greater burden of unmet mental health needs. Tan et al. also suggested that there are ethnic variations in the associations between religion and mental health (13). Han, as the most populous nation in China, has many internal branches. Hakka is a branch of the Han ethnic group, and is one of the far-reaching sub-nations that had wide distribution in the history of China (19). The Chinese characters for Hakka literally mean “guest families”. Unlike other Han Chinese groups, the Hakkas are not named after a geographical region. There are about 80 million Hakka in the world, mainly distributed in Southeast Asian countries and European and American countries. In China, there are about 50 million Hakka live in the junction of Fujian, Guangdong, and Jiangxi provinces. Compared with the general elderly, the Hakka elderly's unique demographic characteristics and life style may have an influence on the mental health status.

However, there is limited study that addresses the mental health of the Hakka elderly. Therefore, this study aims to explore the mental health status of the Hakka elderly in Fujian and to identify its potential socio-demographic correlates by using the Chinese version Symptom Checklist-90-R (SCL-90-R). The findings will not only increase social attention toward the mental health of the Hakka elderly, but also provide baseline information useful for the local government to develop more targeted interventions.

## Materials and methods

### Study design and participants

The present study is a cross-sectional, community-based survey study conducted in Ninghua, Fujian, commonly known as the cradle of the Hakka. Data collection for the present study was nested in a larger cross-sectional population-based survey named the China's Health-Related Quality of Life Survey for Older Adults 2018 (CHRQLS-OA 2018) (20). We collected data on the socio-ecological factors and mental health status of the Hakka elderly during the Spring Festival holidays in 2018. Theoretically, this study requires at least 385 for sample size using 95% confidence level and 5% margin of error. Our aim was to collect no less than 1,000 valid samples. In order to get more enrollments during the specific period of data collection time, the convenience sampling strategy was adopted in this study. Our potential study subjects were residents aged 60 years or older, had a local household registration, and voluntarily participated in the survey. However, those who had a critical illnesses like aphasia, deafness, blindness, paraplegia, etc., had severe mental disorders or had been diagnosed with dementia, or had a history of mental illness were excluded from our target respondents. Considering the low education level of the elderly aged 60 or above in China, a face-to-face interview style was adopted in this study. For those with a poor reading and response abilities, we asked their family caregivers for help. We distributed a total of 1,500 paper questionnaires, but some potential participants refused to participate in the study at the very beginning, and some quit during the interview. Hence, the present study only included 1,262 valid samples in the final analysis with a valid survey response rate of 84.13%.

### Measures

In this study, individual demographic characteristic variables of the participants included sex, age, marital status, Hukou, current residence, education level, average annual household incomes (Chinese Yuan, CNY), survival of parents, living arrangement, and self-rated health status. Age was grouped into five categories namely 60–64, 65–69, 70–74, 75–79, and ≥80. Participants were divided into three subgroups based on their marital status, namely, married/ cohabitation, widowed, and others (unmarried/ divorced, separated, etc.). The setting of alternative options for participants to answer their sex, educational levels, Hukou, current residence, annual household incomes, survival of parents, living arrangement, and self-rated health status are similar to the China Health and Retirement Longitudinal Study (CHARLS)—a nationally representative survey conducted by the National School of Development of Peking University (21).

The present study employed the Chinese version Symptom Checklist-90-R (SCL-90-R) questionnaire to assess the psychological health status of the Hakka elderly in Fujian. The Chinese version SCL-90-R questionnaire was identified as having the good internal consistency and reliability, and was widely used for screening and measuring the progress and outcome of psychological health conditions in China (22). The Chinese version SCL-90-R can evaluate a broad range of psychological problems and symptoms of psychopathology, including somatization (SOM), obsessive-compulsive symptoms (OCS), interpersonal sensitivity (INTS), depression (DEPR), anxiety (ANX), hostility (HOS), phobic anxiety (PHOA), paranoid ideation (PARI), psychoticism (PSY), and sleeping & eating with 90 questions. All questions utilized a five-point Likert scale from 0 (not at all) to 4 (extremely). The higher the score of the Chinese version SCL-90-R indicates greater risk for mental health issues.

### Statistical analyses

Statistical Package for the Social Sciences (SPSS) version 23.0 for Windows (IBM Corporation, Armonk, NY, USA) software was employed to perform all statistical analysis work. The alpha level was set at 5 % to determine statistical significance.

The demographic characteristics of the study sample were summarized in terms of frequencies and proportions. The total scores of SCL-90-R were calculated in terms of means and standard deviations (S. D) by demographic characteristics to assess the distribution of the psychological health status of the Hakka elderly in Fujian across key demographic characteristics. We used *t*-test or one-way analysis of variance (ANOVA) to compare the differences for the total scores of SCL-90-R in different population subgroups. We also used *t*-test to compare the differences for 10 dimensions of SCL-90-R scores between the Chinese general population and the Hakka elderly in this study (23). Furthermore, multivariate analysis were performed by using linear regression analysis to identify the main socio-demographic factors that were most predictive of the total score of SCL-90-R in the Hakka elderly.

### Ethical statement

The study was conducted in accordance with the Declaration of Helsinki. Data collection for the present study was nested in the CHRQLS-OA 2018 led by Wuhan University (20). Hence, the study protocol was reviewed and approved by the Institutional Review Board of School of Health Science and Faculty of Medical Sciences, Wuhan University (IRB Number: 2019YF2050). Informed consent information was included with each paper questionnaire and introduced before the surveys. The participants were guaranteed no risk being involved in participating in the survey, and only those agreed to participate were interviewed.

## Results

### Demographic characteristics of the study sample

A total of 1,262 Hakka elderly people in Fujian participated in this survey study. Among these participants, 48.57% of them were male. The respondents were classified into five age groups including 60–64, 65–69, 70–74, 75–79, and ≥80, accounting for 28.21, 19.65, 17.99, 16.16, and 17.99% of the total sample, respectively. Around two-thirds of the sample hold an agricultural Hukou, and 37.88% were currently living in village. Half of the participants (53.41%) were illiterate. There were 27.65% of the participants earned an average annual household income from 15,000 to 30,000 CNY, and 26.86% earned from 30,000 to 45,000 CNY. Most of the participants (66.79%) were married or in cohabitated with others. There were 58.72% of the participants' with both parents died. Of the participants, 8.24% were living alone, 31.69% were living with spouse only. More than half of the respondents (56.34%) self-rated their own health status as general. The *t*-tests and one-way ANOVA showed that there are statistical differences between participants' gender, age, Hukou, current residence, education level, average household incomes, living arrangement and self-rated health status (all *P* < 0.001). General demographic characteristics of the study sample and SCL-90-R mean scores among different subgroups are summarized in Table 1.

**Table 1:** Participants' demographic characteristics and the total scores of SCL-90-R (*n* = 1,262).

**Variables**	**Categories**	***n*** **(%)**	**Mean** ±**S. D**	***P*** **-value**
Sex	Male	613 (48.57)	1.25 ± 0.21	<0.001
	Female	649 (51.43)	1.31 ± 0.31	
Age (years)	60–64	356 (28.21)	1.43 ± 0.37	<0.001
	65–69	248 (19.65)	1.21 ± 0.09	
	70–74	227 (17.99)	1.27 ± 0.32	
	75–79	204 (16.16)	1.18 ± 0.17	
	≥80	227 (17.99)	1.23 ± 0.14	
Marital status	Married/cohabitation	843 (66.80)	1.27 ± 0.28	0.016
	Widowed	291 (23.06)	1.29 ± 0.26	
	Others	128 (10.14)	1.34 ± 0.29	
Hukou	Agricultural	787 (62.36)	1.32 ± 0.27	<0.001
	Non-agricultural	475 (37.64)	1.22 ± 0.27	
Current residence	Village	478 (37.88)	1.33 ± 0.28	<0.001
	Town	274 (21.71)	1.33 ± 0.32	
	County	510 (40.41)	1.21 ± 0.23	
Education level	Illiterate	674 (53.41)	1.32 ± 0.28	<0.001
	Literacy class/home school	192 (15.21)	1.19 ± 0.15	
	Primary school	192 (15.21)	1.22 ± 0.21	
	≥ Junior high school	204 (16.16)	1.29 ± 0.36	
Average annual household incomes (CNY)	15,000 or lower	260 (20.60)	1.36 ± 0.33	<0.001
	15,001 ~ 30,000	349 (27.65)	1.32 ± 0.27	
	30,001 ~ 45,000	339 (26.86)	1.23 ± 0.24	
	45,001 ~ 60,000	214 (16.96)	1.21 ± 0.23	
	60,001 or higher	100 (7.92)	1.29 ± 0.28	
Survival of parents	Both died	741 (58.72)	1.26 ± 0.24	0.002
	Father or/and mother alive	521 (41.28)	1.31 ± 0.32	
Living arrangement	Living alone	104 (8.24)	1.29 ± 0.29	<0.001
	Living with spouse only	400 (31.70)	1.24 ± 0.24	
	Living with children	435 (34.47)	1.29 ± 0.30	
	Mixed habitation	235 (18.62)	1.32 ± 0.27	
	Others	88 (6.97)	1.37 ± 0.28	
Self-rated health status	Very good/good	374 (29.64)	1.26 ± 0.26	<0.001
	General	711 (56.34)	1.27 ± 0.27	
	Very poor/poor	177 (14.03)	1.38 ± 0.34	

*CNY, China Yuan*.

### The Hakka elderly' scores in dimensions of SCL-90-R

Table 2 records 10 dimensions of SCL-90-R scores in the Hakka elderly in Fujian, and it illustrates that the scores of somatization (1.78 ± 0.55 vs. 1.40 ± 0.46, *P* < 0.001) and phobic anxiety (1.21 ± 0.36 vs. 1.17 ± 0.31, *P* < 0.001) for the Hakka elderly in Fujian appeared to be significantly higher than the Chinese norm, suggesting a higher risk in both types of mental health problems. The respondents' scores in other eight dimensions of SCL-90-R were significantly lower than the national norm (all *P* < 0.001), indicating a lower risk for these specific mental health issues.

**Table 2:** Assessment results of the respondents' scores in all dimensions of SCL-90-R.

**Dimensions**	**Reference range of China (**$\bar{x}$ ±**s)**	**Respondents' scores in this study (**$\bar{x}$ ±**s)**	***t***	***P***
SOM	1.40 ± 0.46	1.78 ± 0.55	24.67	<0.001
OCS	1.59 ± 0.56	1.19 ± 0.35	−38.86	<0.001
INTS	1.38 ± 0.45	1.19 ± 0.27	−23.60	<0.001
DEPR	1.36 ± 0.44	1.17 ± 0.28	−23.31	<0.001
ANX	1.29 ± 0.40	1.19 ± 0.29	−11.50	<0.001
HOS	1.35 ± 0.44	1.13 ± 0.28	−26.86	<0.001
PHOA	1.17 ± 0.31	1.21 ± 0.36	4.21	<0.001
PARI	1.30 ± 0.42	1.17 ± 0.30	−14.56	<0.001
PSY	1.24 ± 0.35	1.19 ± 0.29	−5.33	<0.001
Sleeping and eating	1.52 ± 0.59	1.34 ± 0.48	−12.81	<0.001

*SOM, Somatization; OCS, Obsessive-Compulsive Disorder; INTS, Interpersonal Sensitivity; DEPR, Depression; ANX, Anxiety; HOS, Hostility; PHOA, Phobic Anxiety; PARI, Paranoid Ideation; PSY, Psychoticism*.

### Factors affecting mental health of the Hakka elderly

Table 3 presents the results of multivariate analysis of factors affecting mental health of the Hakka elderly. The results suggested that the Hakka females had a higher total score of SCL-90-R than males (β = 0.030, 95% CI = 0.001 to 0.060). When compared with individuals aged 60–64, those aged 65–69 (β = −0.222, 95% CI = −2.267 to −0.178), those aged 70–74 (β = −0.145, 95% CI = −0.193 to −0.097), those aged 75–79 (β = −0.243, 95% CI = −0.296 to −0.190), and those aged 80 or above (β = −0.224, 95% CI = −0.278 to −0.169) had a lower total score of SCL-90-R, indicating a lower risk of suffering from mental health problems. The results confirmed that those widowed elderly people had a worse mental health condition with a higher total score of SCL-90-R (β = 0.053, 95% CI = 0.008 to 0.097). The Hakka elderly who were currently living in the town (β = −0.091, 95% CI = −0.132 to −0.050) displayed a lower total score of SCL-90-R. In terms of education level, these who had experience of literacy class/ home school (β = −0.071, 95% CI = −0.120 to −0.021) and primary school (β = −0.061, 95% CI = −0.115 to −0.006) were detected as lower scores than these who were illiterate. Compared with individuals with an average annual household incomes <15,000 RMB, those who were classified with 30,001 ~ 45,000 CNY (β = −0.028, 95% CI = −0.068 to 0.013), 45,001 ~ 60,000 CNY (β = −0.103, 95% CI = −0.164 to −0.042) and more than 60,000 CNY (β = −0.138, 95% CI = −0.214 to −0.062) had a lower total score of SCL-90-R. Participants with father or/ and mother alive (β = 0.047, 95% CI = 0.009 to 0.085) exhibited a higher total score of SCL-90-R than these with parents both died. With “living alone” as the reference group, people in “living with spouse only” (β = −0.075, 95% CI = −0.142 to −0.007) and “living with children” groups (β = −0.104, 95% CI = −0.169 to −0.040) displayed a lower score of SCL-90-R, but, people in “mixed habitation” (β = 0.100, 95% CI = 0.039, 0.160) and “others” groups (β = 0.123, 95% CI = 0.047 to 0.198) displayed higher scores of SCL-90-R. Those with a very poor/ poor self-related health status (β = 0.110, 95% CI = 0.058 to 0.162) also scored higher.

**Table 3:** Predictors of factors associated with the total score of SCL-90-R among the Hakka elderly.

**Variables**	**Categories**	β	**S. E**	***t***	***P*** **-value**	**95% CI for** β
Sex (ref = male)	Female	0.030	0.015	2.019	0.044	(0.001, 0.060)
Age (ref = 60–64)	65–69	−0.222	0.022	−9.907	<0.001	(−2.267, −0.178)
	70–74	−0.145	0.025	−5.903	<0.001	(−0.193, −0.097)
	75–79	−0.243	0.027	−9.040	<0.001	(−0.296, −0.190)
	≥80	−0.224	0.028	−8.053	<0.001	(−0.278, −0.169)
Marital status (ref = married/cohabitation)	Widowed	0.053	0.023	2.319	0.021	(0.008, 0.097)
	Others	0.047	0.027	1.739	0.082	(−0.006, 0.100)
Hukou (ref = agricultural)	Non-agricultural	−0.009	0.026	−0.336	0.737	(−0.059, 0.042)
Current residence (ref = village)	Town	−0.091	0.021	−4.356	<0.001	(−0.132, −0.050)
	County	0.028	0.029	0.972	0.331	(−0.029, 0.086)
Education level (ref = illiterate)	Literacy class/home school	−0.071	0.025	−2.796	0.005	(−0.120, −0.021)
	Primary school	−0.061	0.028	−2.189	0.029	(−0.115, −0.006)
	≥ Junior high school	0.012	0.030	0.408	0.683	(−0.047, 0.072)
Average annual household incomes (ref = 15,000 or lower)	15,000 ~ 30,000	−0.028	0.021	−1.329	0.184	(−0.068, 0.013)
	30,001 ~ 45,000	−0.087	0.026	−3.342	0.001	(−0.138, −0.036)
	45,001 ~ 60,000	−0.103	0.031	−3.294	0.001	(−0.164, −0.042)
	60,001 or higher	−0.138	0.039	−3.550	<0.001	(−0.214, −0.062)
Survival of parents (ref= both died)	Father or/and mother alive	0.047	0.019	2.431	0.015	(0.009, 0.085)
Living arrangement (ref = living alone)	Living with spouse only	−0.075	0.035	−2.167	0.030	(−0.142, −0.007)
	Living with children	−0.104	0.033	−3.170	0.002	(−0.169, −0.040)
	Mixed habitation	0.100	0.031	3.225	0.001	(0.039, 0.160)
	Others	0.123	0.039	3.175	0.002	(0.047, 0.198)
Self-rated health status (ref = very good/good)	General	0.021	0.018	1.130	0.259	(−0.015, 0.056)
	Very poor/poor	0.110	0.026	4.182	<0.001	(0.058, 0.162)

## Discussion

In this study, we found that the SCL-90-R score for 10 dimensions obtained from the Hakka elderly was different from the Chinese national norm. Specifically, eight dimensions were scored lower than the national norm, which suggests that the mental health status of the Hakka elderly in Fujian is better than the national level. But the Hakka elderly in Fujian were more likely to have somatization and phobic anxiety compared with national level. Previous study have reported a higher score in phobic anxiety among the Hakka population (24). The truth is physiological function declines with age, and the difference in somatization is easy to understand since the average age of Hakka elderly in Fujian is higher than the norm (23). Moreover, economic and social development are most important determinants of physical health. People with lower socioeconomic status generally have lower health levels, while the Hakka elderly in Fujian are mainly distributed in less developed areas in Fujian, and 37.8% of the participants in this study come from rural areas. Chase et al.'s study also found that due to cultural differences, people in different regions have different mental status (25), and the Hakkas received a higher score in phobic anxiety may also due to cultural factors that the Hakkas tend to advocate defense strategies with more tolerant of fear.

Previous studies had shown that the mental health of the elderly is related to some demographic factors, such as gender, marriage, education, and social activities (26, 27). In the study, we found that the Hakka female elderly were more likely to suffer from mental illness than males, which is consisted with other studies (28, 29). Mental health is positively associated with social support and social network (29, 30), as the disadvantaged group, women in China, especially for the elderly, are generally poorly educated and mainly play the role of housewives. They tend to have narrow social networks, few social activities and limited social capital as compared with males (31).This is most evident among the Hakka female groups according to Chen's study. Besides, marital status has long been recognized as one of the most important social determinants of health. Women's mortality advantage contributes to more life years, and they are more likely to be widowed. In the present study, the widowed Hakka elderly were detected to have worse mental health conditions. This finding also echoes the previous studies (32–34). Result from a cross-sectional study conducted in Southwest China pointed out that losing the spouse might lead to an increased risk of mental health problems, such as loneliness and depression (25). Under the one-child policy, the loneliness and desolation could cause many mental health issues, especially when the left-behind seniors in rural areas lost their spouses and have to live alone. Therefore, more humanistic care and social support is needed to protect this special population.

Among the participants, people aged 60–64 were more likely to suffer from mental issues, which seemed inconsistent with the previous studies (26, 35, 36). A cross-sectional study showed that with the increase of age, the demand for long-term service may bring the elderly a greater economic burden and the burden of being taken care of (26). But the other point of view is that the younger elderly have to face the big change and transition of social roles and the decline of socio-economic status because of the retirement (37). Besides, they may commonly have more social responsibility such as the burden of taking care of their parents or grandchildren, while the oldest individuals do not have these concerns in China. Similar to earlier findings (38), those who were living with spouse only or living with children have a better mental health. It is well-understood that living with children provides a higher level of social support.

Interestingly, this study found that the elderly with parents both died had a better mental health condition as compared with the elderly with father or/and mother alive. This finding seems at odds with our understanding. But it is similar to the research results of Abdul et al.'s study (39). The possible mechanism might be that the elderly with father or/ and mother alive have the responsibility (financial and mental pressure) of taking care of the old parent(s). Consistent with the conclusions reported by many previous studies (40, 41), our study also indicated that the Hakka elderly with low average annual household incomes had a worse state of mental wellbeing. The same conclusion applies to people with low level of education. Although it is understandable that people with low economic and educational level usually show much less ability to access and utilize appropriate mental health care delivery resources (34). There are some related studies targeting the Chines elderly had come to different conclusions (38, 42). It seems to be inconclusive regarding the relationship between the income level and the mental health among the elderly in China. Since China is undergoing dramatic social changes with the rapid economic growth in the past few years, related further research is needed.

## Conclusion

In conclusion, the present study has gained an overall understanding upon the mental health of the Hakka elderly in Fujian. The results of this study have clearly identified that the scores in eight dimensions of SCL-90-R were significantly lower than the national norm, suggesting a healthier mental health conditions of the Hakka elderly in Fujian. However, the worse mental health conditions of the Hakka elderly in somatization and phobic anxiety were detected with significantly higher scores than the national norm. The overall mental health status was shown to be worse among those who were female, widowed, living in village, and those with low education and low average annual household incomes, those with father or/ and mother alive. Hence, the Hakka elderly with the characteristics mentioned above should be regarded as the most vulnerable groups, and more attention should be paid. We recommend that caregivers, mental health administrators, health policy makers should target the most vulnerable populations and develop the personalized health promotion measures, interventions, or support initiatives based on the real condition of the elderly.

To the best of our knowledge, this study is among the first targeting the Hakka elderly to reveal their mental health conditions and associated contributing factors. The following limitations of the present study should be noted: Firstly, only correlation rather than causal relationship was explored due to the cross-sectional design of this study. Hence, longitudinal survey on the influencing factors of mental health are needed. Secondly, the sampling of individuals was not random. The present study can only understand the current the status of, and factors associated with mental health among Hakka elderly in Fujian, China. However, the Hakka people are also mainly distributed in Guangdong and Jiangxi provinces of China, and other Southeast Asian countries like Thailand, Malaysia, Singapore. Our samples cannot well summarize the general characteristics of the Hakka elderly. Therefore, the conclusions of this study need to be cautious when extrapolating. Moreover, non-response bias was not assessed, as only those agreeing to participate were included in study analyses. In addition, some socio-demographic factors (e.g., occupation before retirement) may also related to mental health status variables of the elderly, were not included in the data analysis. For future studies, research on the mental status of the Hakka elderly in different regions or case-control studies with other populations (e.g., general population or non-Han population) should be performed.

## Data availability statement

The original contributions presented in the study are included in the article/supplementary material, further inquiries can be directed to the corresponding author/s.

## Ethics statement

The study was conducted in accordance with the Declaration of Helsinki, and the study protocol was reviewed and approved by the Institutional Review Board of School of Health Science and Faculty of Medical Sciences, Wuhan University (IRB Number: 2019YF2050). Informed consent information was included with each paper questionnaire and introduced before the surveys. The participants were guaranteed no risk being involved in participating in the survey, and only those who agreed to participate were interviewed.

## Author contributions

XL and WW conceived this research. XL was responsible for the methodology. XL, FL, and WR conducted software analyses, gathered resources, curated all data, wrote and prepared the original draft, and were responsible for project administration. YC conducted necessary validations. XL, SQ, and WW conducted a formal analysis and managed the investigation. XL, WR, and WW reviewed and edited the manuscript, were responsible for visualization, supervised the project, and acquired the funding. All authors contributed to the article and approved the submitted version.

## Funding

The present study was mainly supported by Fujian Basic Theory Research Foundation of Philosophy & Social Science Guided by Marxism (Grant No. JSZM2021027). Meanwhile, this work was also partially supported by Fujian Medical University's high-level talent research start-up project (Grant No. XRCZX2020020) and the National Natural Science Foundation of China (Grant Nos. 71673121 and 71373102).

## Conflict of interest

The authors declare that the research was conducted in the absence of any commercial or financial relationships that could be construed as a potential conflict of interest.

## Publisher's note

All claims expressed in this article are solely those of the authors and do not necessarily represent those of their affiliated organizations, or those of the publisher, the editors and the reviewers. Any product that may be evaluated in this article, or claim that may be made by its manufacturer, is not guaranteed or endorsed by the publisher.
